# Draft Genome Sequence of a Thermophilic Cyanobacterium from the Family *Oscillatoriales* (Strain MTP1) from the Chalk River, Colorado

**DOI:** 10.1128/genomeA.01571-15

**Published:** 2016-02-18

**Authors:** Patrick C. Hallenbeck, Melanie Grogger, Megan Mraz, Donald Veverka

**Affiliations:** Department of Biology, Life Sciences Research Center, U.S. Air Force Academy, Colorado Springs, Colorado, USA

## Abstract

The draft genome (57.7% GC, 7,647,882 bp) of the novel thermophilic cyanobacterium MTP1 was determined by metagenomics of an enrichment culture. The genome shows that it is in the family *Oscillatoriales* and encodes multiple heavy metal resistances as well as the capacity to make exopolysaccharides.

## GENOME ANNOUNCEMENT

Cyanobacteria have had a major influence on the earth for at least the last 2.95 billion years (1). Although cyanobacteria have been described and studied for over 150 years, surprisingly their diversity is only sparsely described at the genomic level (2). Indeed, it has been estimated that a great deal of cyanobacterial metabolic diversity remains to be discovered, with obvious consequences for biotechnology and ecological understanding (3). Cyanobacteria are of enormous ecological importance and have managed to colonize, in either free-living or symbiotic forms, most of the ecological niches available on the earth (4–7).

The MTP1 draft genome was 57.7% GC and consisted of 7,647,882 bp. Surprisingly, MTP1 appears by phylogenomics to be only relatively distantly related to known cyanobacteria, with its closest relatives, *Geitlerinema* and *Leptolyngbya*, in the family *Oscillatoriales*. The rRNA from MTP1 was somewhat related to these two genera: *Geitlerinema* sp. PCC 7407 SSU RNA 92% (1362/1486), LSU RNA 90% (2608/2898); and *Leptolyngbya boryana* SSU RNA 90% (1345/1488) (90%), LSU RNA 88% (2564/2910). The draft genome consisted of 6,695 coding sequences and 81 RNAs, as determined by RAST and SEED (8, 9). Again, of these coding sequences relatively few were highly similar to their orthologs in either cyanobacterium. With *Geitlernema* sp. PCC 7407 only 27 were >90% identical, and only 167 were >80% identical. With *Leptolyngbya borana* only 21 were >90% identical, and only 151 were >80% identical. Thus, the MTP1 draft genome is intrinsically of interest in providing additional insight into cyanobacterial diversity. In addition, further analysis is sure to yield information on specific niche adaptations and ecological interactions. For example, the MTP1 genome encodes a variety of resistance systems (over 84 genes), in particular to heavy metals, including copper, cobalt, cadmium, zinc, mercury, and arsenic. As well, it encodes 87 genes involved in capsule and extracellular polysaccharide (EPS) synthesis, suggesting their possible importance in mat formation and interactions with other organisms. EPS may have interesting biotechnological properties (10, 11) and play diverse roles in nature in promoting adherence (12), formation of microbial mats (13–15), and allowing for long-term survival under adverse conditions (16). The genome also encodes proteins related to the capacity for fermentative metabolism, including several hydrogenases.

Genomic DNA was isolated from an enrichment culture of a sample obtained at a thermal source at Chalk Creek, Colorado. The library was prepared using a Nextera DNA sample preparation kit (Illumina) following the manufacturer’s user guide, with subsequent simultaneous fragmentation and addition of adapter sequences by a limited-cycle (5 cycles) PCR. The final library concentration (1.60 ng/µL) was measured using the Qubit dsDNA HS assay kit (Life Technologies), and the average library size (883 bp) was determined using the Agilent 2100 Bioanalyzer (Agilent Technologies). The library was sequenced by using a 600 Cycles v3 reagent kit (Illumina) in MiSeq (Illumina). Assembly was performed by MR DNA (Shallowater, TX) using NGEN (DNAstar) as the primary assembly method followed by manual optimization.

### Nucleotide sequence accession numbers.

This whole-genome shotgun project has been deposited at DDBJ/EMBL/GenBank under the accession number LNAA00000000. The version described in this paper is version LNAA01000000.
